# Supplementary material for: Japanese Honeybees (Apis cerana japonica Radoszkowski, 1877) May Be Resilient to Land Use Change
Source: Insects. 2021 Jul 30;12(8):685. doi: 10.3390/insects12080685 (PMC8396638; doi:10.3390/insects12080685)
Supplement: Supplementary file 1 [file insects-12-00685-s001.zip › Appendix 1.pdf]

APPENDIX 1

Pollen biotype identification

Biotype classifications and descriptions used to identify pollen grains found in samples collected from five *Apis cerana japonica* hives in Nagasaki-ken, Japan, between June and September 2019. Potential species names are also given where possible, based on pollen samples collected directly from flowering plants and a palynomorph guide of Japanese flora by Shimakura (1973). Reference images are approximately relatively sized.

Number	Possible species name(s)	Description	Reference Image
1	*Aralia elata*	Small, circular/semi-circular	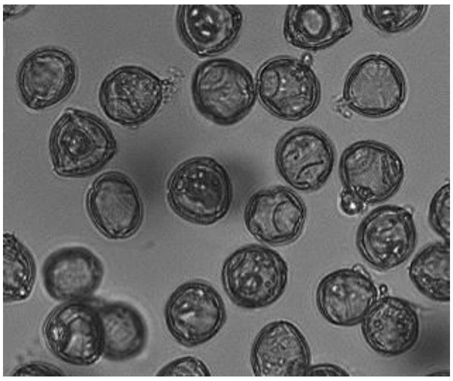
2		Large, asymmetrical, pale, slightly dotted	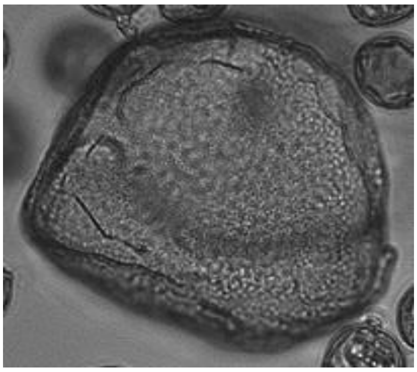
3	*Firmiana simplex*	Medium, dark, 3-way symmetry, rounded	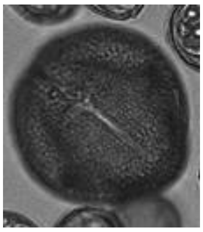

4		Medium, oval, asymmetrical, dotty. Often burst	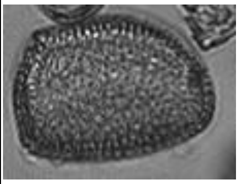 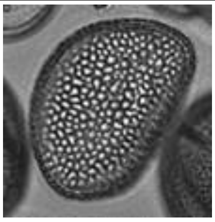 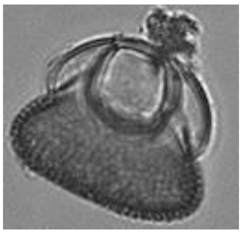
5		Small, triangular, circles on points	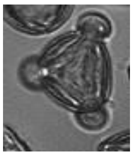
6		Medium, dark, circular	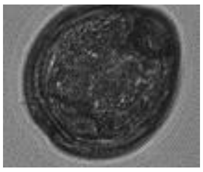
7		Medium-small, 3-way symmetry, semi-angular, internal lines, darkish	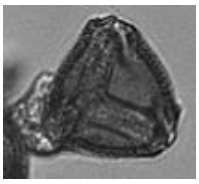
8	*Abelia* (a.k.a. *Linnaea*) x *grandiflora*	Medium-small, grainy, dark, circular/semi-circular. Sometimes has three circles on corners	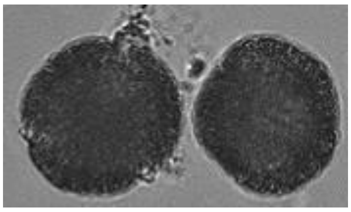
9	*Fraxinus griffithii*	Medium-small, circular/semi-circular, darkish, border	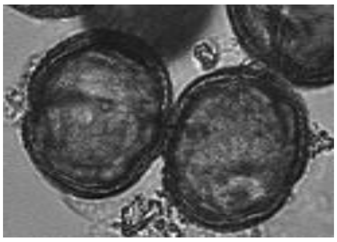

10		Very small, light, circular/semi-circular.	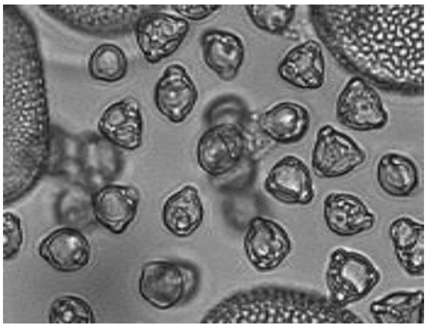
11		Medium-small, oval, light, lines down. Coffee bean-like. 3-way symmetry.	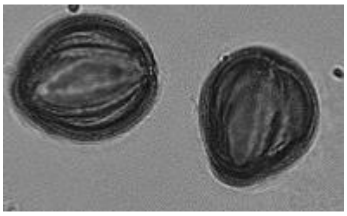 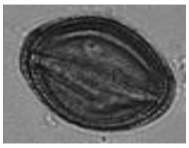
12	*Citrus/Fortunella crassifolia*<br>*Firmiana simplex*	Like 3, but medium-small, semi-circular.	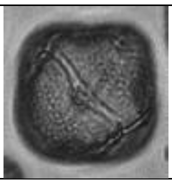 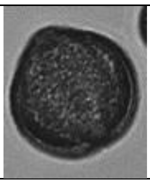
13	*Lagerstroemia indica*	Medium, asymmetrical, slightly pointed	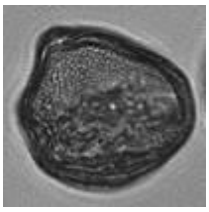
14		Medium, oblong, slightly pointed at ends, arcing lines through	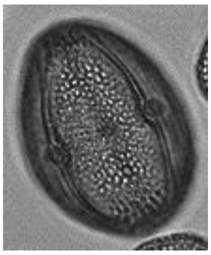 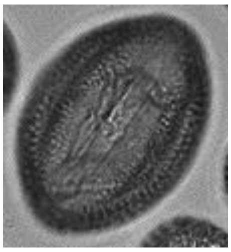
15	*Trifolium repens*	Like 3, but lighter and a bit pointed on sides	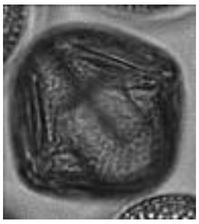

16	*Dendropanax trifidus*<br>*Euonymus japonicus*	15, but medium-small	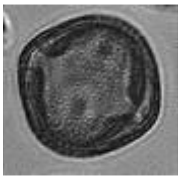
17	*Lithocarpus edulis*	Small, oval, lines arcing through.	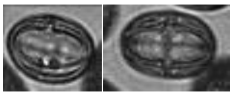
18		Like 5 but medium, semi-angular.	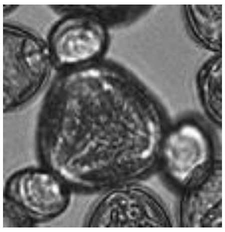
19		Medium, semi-circular, darkish middle, light circles on corners	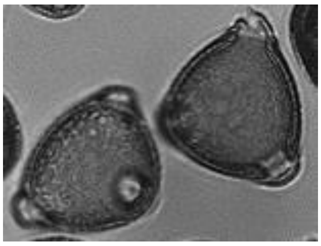
20	*Mallotus japonicus*<br>*Citrus/Fortunella crassifolia*<br>*Dendropanax trifidus*<br>*Euonymus japonicus*	Similar to 16, but more indents, elongated. From end on: small-medium, three rounded sides, triangle inside, with points between indents.	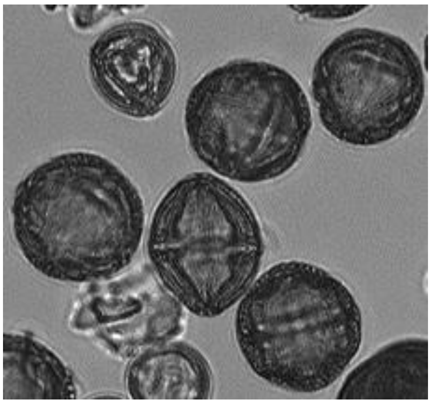<br>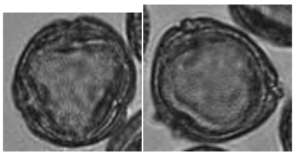
21		Medium-small, circle with triangle inside, or diamond from side on	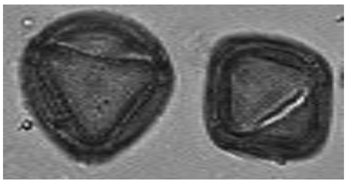

22	*Dendropanax trifidus*	Medium-small, asymmetrical diamond, with internal lines. Sharp triangular from end-on	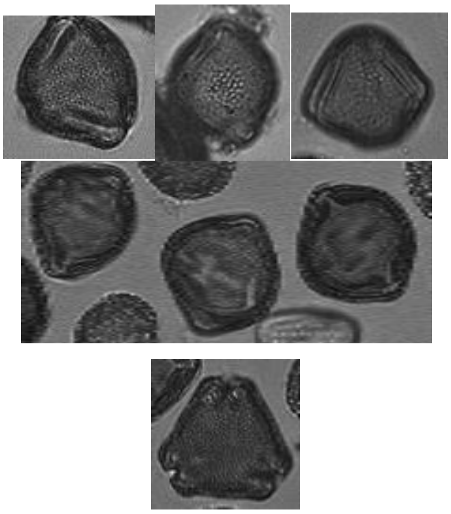
23	*Vitis ficifolia* var. *lobata*	Small-medium, circular/semi-triangular, border, granular inside	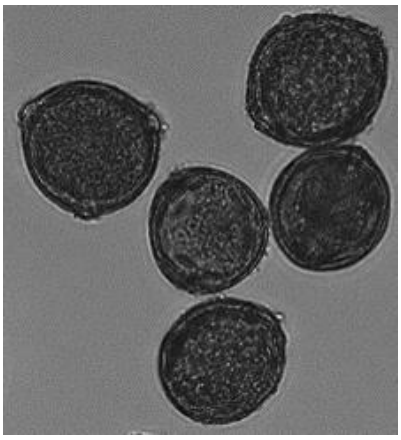
24		Medium, oblong, slightly pointed at sides, lines through with little dents	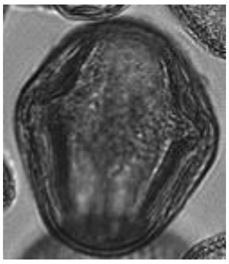
25		Small, pale, simple, oval, internal lines	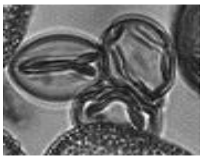
26		Medium, very light, cell-like, dotted randomly inside, thin border	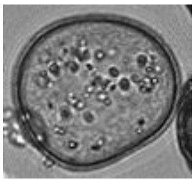

27		Medium, circular, granular, thin border	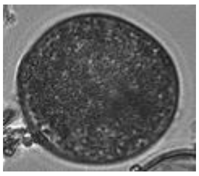
28		Like 27, but small	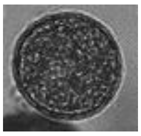
29		Medium-small, mottled inside, darker wiggly border, circular	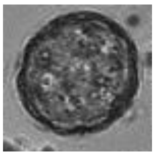
30		Medium-small, triangle with concave sides inside, lobed (inter-semiangular)	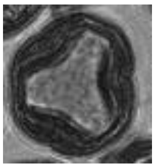
31		Medium-small, semi-circular, Y shape joining indents sometimes visible	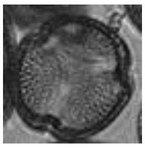
32		Medium-small, very wiggly outline, darkish	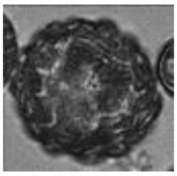
33		Medium-small, slightly wiggly circular, grainy inside	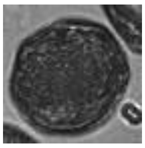
34		Like 3, but slightly asymmetrical and lighter. Like 31 (but medium) when seen end-on.	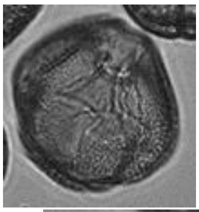 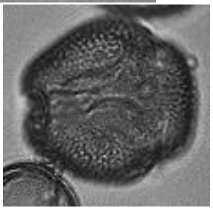

35		Small-medium, semi-circular, sometimes circles at indents where sides meet. Faint triangle inside joining indents.	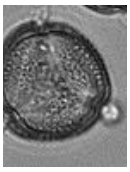
36		Medium, pointed oval, granular and dark inside	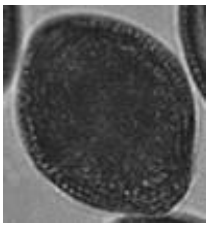
37	*Ternstroemia gymnanthera*	Like 20 but small and pale	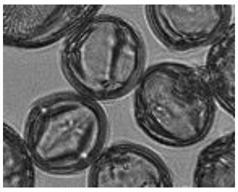 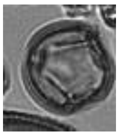
38		Small, like 36 but granular inside inner triangle	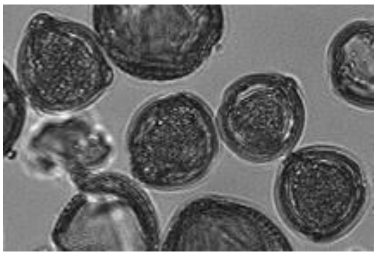
39	*Aralia elata*	Small, like 39 but clear Y connecting points	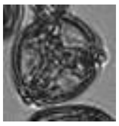
40		Medium-small, pointed oval, lines arcing through	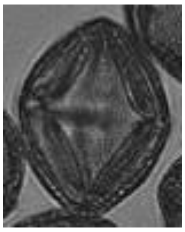
41		Medium-large, dark, granular.	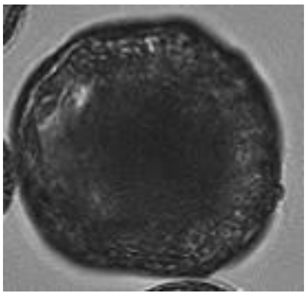

42		Like 26 but very large	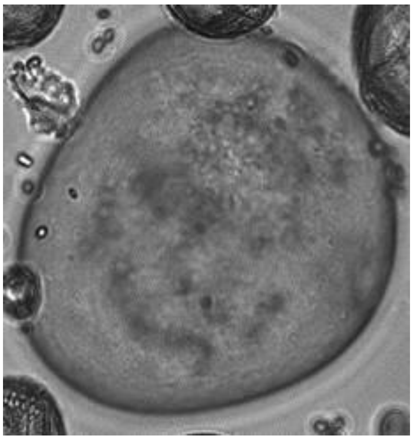
43	*Tetradium daniellii* var. *hupehensis*	Like 8 but small	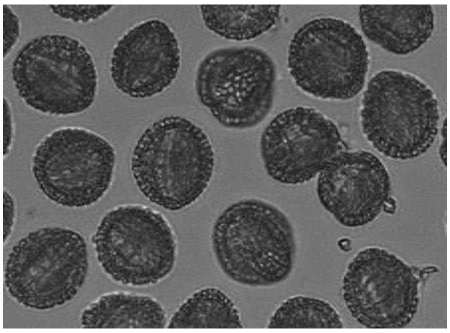
44		Small, light, oval, border, slightly dotted	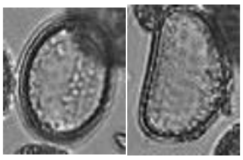
45		Like 3, but small, tri-lobate circular	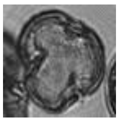
46		Small oval with two circles opposite each other	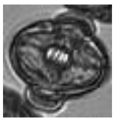
47		Like 20 but medium-small. Darker than 37	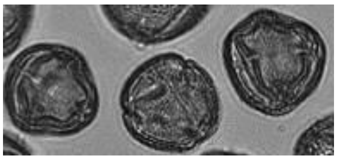
48	*Trifolium repens*	Rounded oval with arcing lines	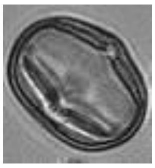
49		Pale triangular	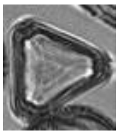

50		Semi-circular with sharp indents at points	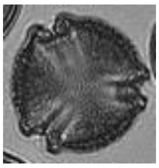
