# Supplementary material for: Japanese Honeybees (Apis cerana japonica Radoszkowski, 1877) May Be Resilient to Land Use Change
Source: Insects. 2021 Jul 30;12(8):685. doi: 10.3390/insects12080685 (PMC8396638; doi:10.3390/insects12080685)
Supplement: Supplementary file 1 [file insects-12-00685-s001.zip › supplementary materials 2 pollen.pdf]

Table S1. Pollen collection dates for *Apis cerana japonica* hives in Nagasaki-ken, Japan.

**Week**	**Date**	**Hive 15**			**Hive 16**			**Hive 19**		**Hive 11**
		Brood	***Trap***	***Basket***	***Brood***	***Trap***	***Basket***	***Brood***	***Trap***	***Brood***
1	13.6.19							✓		✓
2	20.6.19			✓			✓			
2	21.6.19	✓			✓					
3	24.6.19		✓			✓				
3	25.6.19								✓	
3	26.6.19									✓
4	Typhoon weather									
5	12.7.19	✓		✓	✓		✓			
6	16.7.19							✓	✓	✓
6	19.7.19		✓			✓				
7	Typhoon weather									
8	29.7.19	✓			✓					
8	30.7.19		✓			✓				
8	31.7.19			✓			✓			
9	8.8.19									✓

Table S2. The land types used to classify vegetation cover across Nagasaki and Saga provinces of Japan. Rural/urban categorisation used to calculate rural-urban ratios are listed. Vegetation classification and original map labels were sourced from vegetation maps freely available from the Japanese Ministry of the Environment's Biodiversity Centre (Ministry of the Environment, Biodiversity Centre, ND).

Original map label (MAJOR1)	New label	Name (Japanese)	Name (English)	Rural or urban?
6026	1	アカガシーミヤマシキミ群集	Japanese evergreen oak (*Quercus acuta*) and Japanese skimmia (*Skimmia japonica*) crowd	Rural
6030	2	ケヤキ群落	Japanese elm (*Zelkova serrata*) community	Rural
6075	3	スダジイーヤブコウジ群集	Itajii chinkapin (*Castanopsis sieboldii*) and marlberry (*Ardisia japonica*) crowd	Rural
6078	4	ミミズバイースダジイ群集	*Symplocos glauca* and Itajii chinkapin crowd	Rural
6079	5	スダジイーミミズバイ群集	Itajii chinkapin and *Symplocos glauca* population	Rural
6087	6	スダジイーホソバカナワラビ群集	Itajii chinkapin and *Arachniodes exilis* crowd	Rural
6111	7	タブームサシアブミ群集	Cobra lily (*Arisaema ringensi*) crowd	Rural
6123	8	マサキートベラ群集	Japanese spindle (*Euonymus japonicus*) and Japanese pittosporum (*Pittosporum tobira*) crowd	Rural
6128	9	ハマビワーオニヤブソテツ群集	*Litsea japonica (Thunb.) Juss.* and Japanese holly fern (*Cyrtomium falcatum*) crowd	Rural
7009	10	クヌギ群落	Sawtooth oak (*Quercus acutissima*) community	Rural
7017	11	シイ・カシ萌芽林	Chinquapin (*Castanopsis*) and ring-cupped oak (*Cyclobalanopsis*) species seedling forest	Rural
7025	12	マテバシイーハクサンボク群落	Japanese stone oak (*Pasania edulis*) and Japanese viburnum (*Viburnum japonicum*) community	Rural
7036	13	伐跡群落	Logging community	Rural
7047	14	ヤダケーメダケ群落	Arrow bamboo (*Pseudosasa japonica*) and medake bamboo (*Pleioblastus simonii*) community	Rural
7054	15	ススキ群団	Maiden silvergrass (*Miscanthus sinensis*) group	Rural
7065	16	ススキーチガヤ群落	Maiden silvergrass and cogongrass (*Imperata cylindrica*) community	Rural
7081	17	アカマツ群落	Japanese red pine (*Pinus densiflora*) canopy	Rural

7085	18	アカマツーヤマツツジ群集	Japanese red pine and torch rhododendron (*Rhododendron kaempferi*) crowd	Rural
7088	19	アカマツーオンツツジ群集	Japanese red pine and *Rhododendron weyrichii* var. *weyrichii* crowd	Rural
7093	20	クロマツ群落	Japanese black pine (*Pinus thunbergii*) canopy	Rural
8008	21	ヨシクラス	Common reed (*Phragmites australis*) class	Rural
8040	22	砂丘植生	Dune vegetation	Rural
8051	23	ハチジョウススキ群落	Maiden silvergrass community	Rural
8101	24	ハイビャクシン群落	Japanese garden juniper (*Juniperus procumbens*) community	Rural
9016	25	スギ・ヒノキ・サワラ植林	Japanese cedar (*Cryptomeria japonica*), Japanese cypress (*Chamaecyparis obtusa*) and Sawara cypress (*Chamaecyparis pisifera*) plantation	Rural
9017	26	スギ・ヒノキ植林	Japanese cedar and Japanese cypress plantation	Rural
9032	27	クスノキ植林	Camphor tree (*Cinnamomum camphora*) plantation	Rural
9033	28	マテバシイ植林	Japanese stone oak plantation	Rural
9035	29	落葉広葉樹植林	Other plantations (deciduous broad-leaved trees)	Rural
9060	30	モウソウチク林	Moso bamboo *(Phyllostachys edulis*) forest	Rural
9061	31	マダケ・ハチク林	Japanese timber bamboo (*Phyllostachys bambusoides*) *and* white bamboo (*Phyllostachys nigra* var. *henonis*) forest	Rural
9062	32	常緑果樹園	Evergreen orchard	Rural
9064	33	落葉果樹園	Deciduous orchard	Rural
9068	34	茶畑	Tea plantation	Rural
9072	35	畑地雑草群落	Upland weed community	Rural
9077	36	ヒメムカシヨモギーオオアレチノギク群落	Horseweed (*Erigeron canadensis)* and white horseweed (*Conyza sumatrensis*) community	Rural
9082	37	牧草地	Pasture	Rural
9090	38	ゴルフ場、飛行場の芝地	Golf course, airfield turf	Urban
9096	39	ゴルフ場、飛行場	Golf course, airfield	Urban
9098	40	水田雑草群落	Paddy field weed community	Rural
9101	41	休耕田雑草群落	Fallow field weed community	Rural

9902	42	市街地	City area	Urban
9905	43	緑の多い住宅地	Green residential area	Urban
9906	44	公園、墓地	Parks, cemeteries	Urban
9910	45	緑の多い住宅地、公園、墓地	Green residential areas, parks and cemeteries	Urban
9915	46	工場地帯	Factory area	Urban
9919	47	造成地	Land for development	Urban
9927	48	採石場	Quarry	Urban
9929	49	干拓地	Reclaimed land	Urban
9931	50	開放水域	Open water	Rural
9933	51	自然裸地	Natural bare land	Rural
9999	52	現存植生不明区分	Existing vegetation unknown	Rural

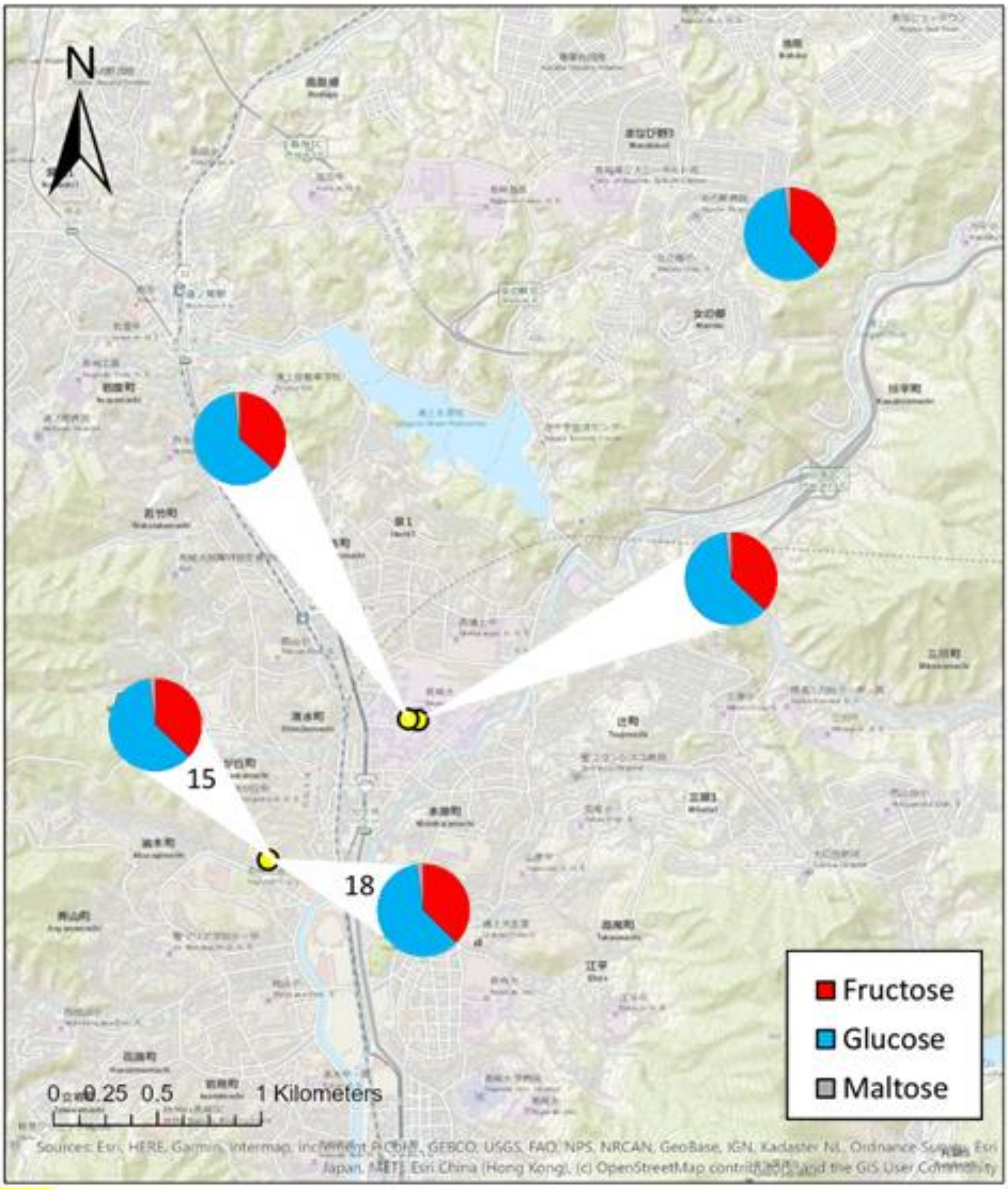

Figure S1. Proportion of fructose, maltose and glucose in honey samples collected from five *Apis cerana japonica* hives in Nagasaki, Japan. Yellow dots represent hives where pie chart could not be placed directly in the correct location. Where multiple hives had the same location, pie charts are labelled with hive number. Sugar proportions were calculated using High Performance Liquid Chromatography.

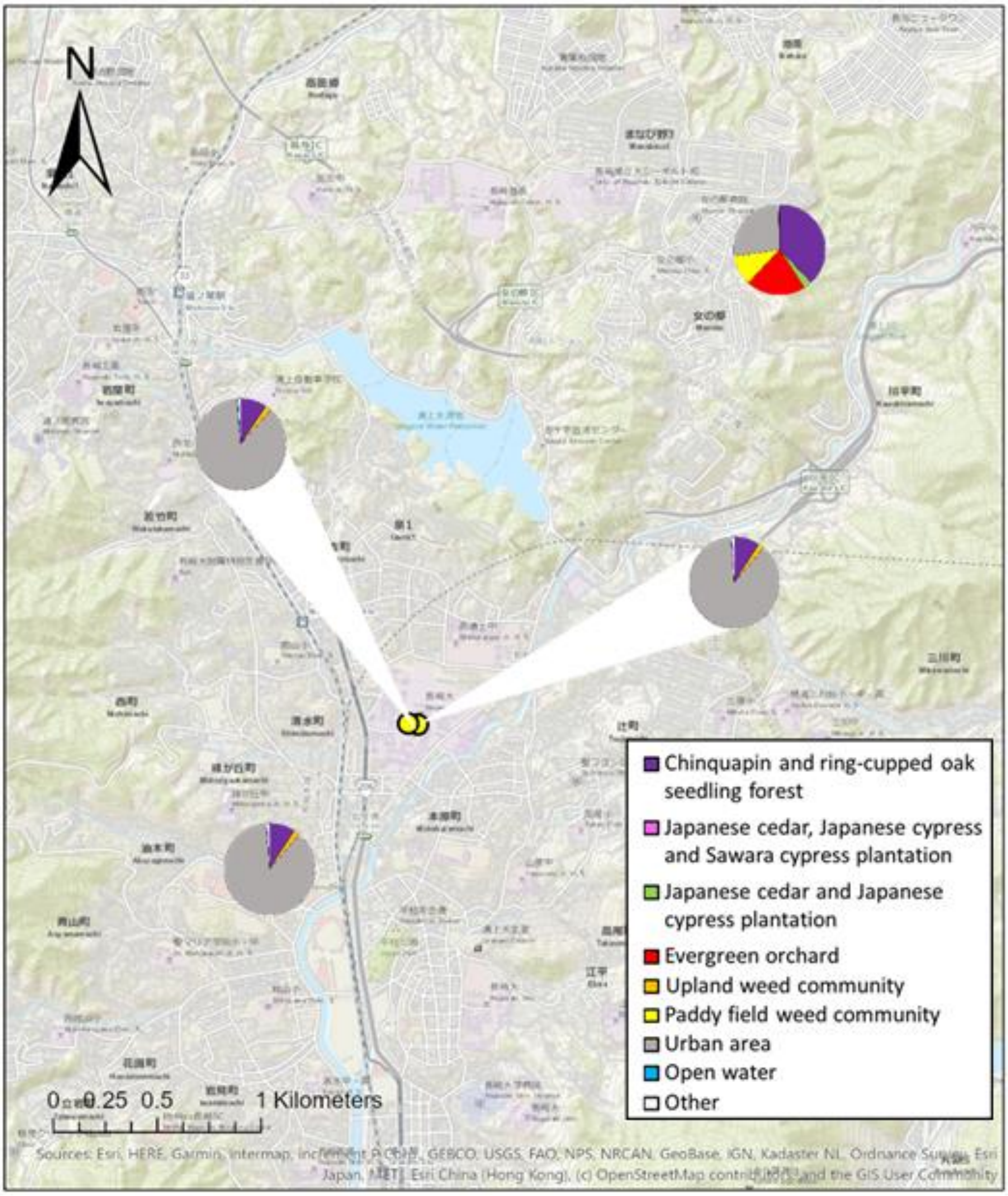

Figure S2. Proportion of land uses in a 1 km radius surrounding five *Apis cerana japonica* hives in Nagasaki, Japan. Land uses which were dominant for at least one hive are shown, along with all other land types, grouped into 'other'. Yellow dots represent hives where pie chart could not be placed directly in the correct location. Hives 15 and 18 were located in the same apiary, so are shown using one pie chart. Land use was calculated using vegetation maps freely available from the Japanese Ministry of the Environment's Biodiversity Centre (Ministry of the Environment, Biodiversity Centre, ND).
